# The shortening of leukocyte telomere length relates to DNA hypermethylation of LINE-1 in type 2 diabetes mellitus

**DOI:** 10.18632/oncotarget.18167

**Published:** 2017-05-22

**Authors:** Yue Wu, Wei Cui, Donghong Zhang, Wei Wu, Zhuo Yang

**Affiliations:** ^1^ Department of Clinical Laboratory, National Cancer Center/Cancer Hospital, Chinese Academy of Medical Sciences and Peking Union Medical College, Beijing, 100021, China; ^2^ Department of Clinical Laboratory, Peking Union Medical College Hospital, Peking Union Medical College and Chinese Academy of Medical Sciences, Beijing, 100730, China; ^3^ Department of Cardiology, The Second Affiliated Hospital of Wenzhou Medical University, Wenzhou 325027, Zhejiang, China; ^4^ Department of Genetics, Albert Einstein College of Medicine, Bronx, NY 10461, USA

**Keywords:** type 2 diabetes mellitus, leukocyte telomere length, DNA methylation, LINE-1, glycated hemoglobin

## Abstract

**Background:**

We aim to investigate the cross-talking of leukocyte telomere length (LTL) and DNA methylation of LINE-1 in type 2 diabetes mellitus (T2DM).

**Results:**

LTL (ratio of the copy number of telomere [T] repeats to that of a single [S] gene) was significantly shortened in T2DM compared with controls (0.94 ± 0.41 vs. 1.14 ± 0.48, *P* < 0.001), and decreased steadily with age in both controls and T2DM. Conversely, significant increase of LINE-1 DNA methylation was found in T2DM compared with controls (49.60 ± 14.55 vs. 37.81 ± 9.07, *P* < 0.001). Moreover, age, HbA1c, and LINE-1 methylation ratio were stably negatively related with LTL after multi-adjustment. Shorter LTL was associated with an increased risk of T2DM [adjusted OR (95% CI) = 2.458 (1.192, 5.070), *P =* 0.015], while lower LINE-1 DNA methylation levels could reduce the risk of T2DM [adjusted OR (95% CI) = 0.189 (0.089, 0.400), *P* < 0.001].

**Materials and Methods:**

We performed a hospital-based case–control study of 205 T2DM patients and 213 subjects of healthy control with sex and age matched. LTL and DNA methylation of LINE-1 was measured by quantitative PCR and quantitative methylation-specific PCR (qMSP), respectively.

**Conclusions:**

Our research demonstrates the association between shorter LTL and LINE-1 hyper-methylation in Chinese T2DM patients. These findings suggest that shorter LTL might be associated with T2DM in a manner dependent of epigenetic level.

## INTRODUCTION

Type 2 diabetes (T2DM) is known as an age-related disease affected by both genetic and environmental factors [1]. Telomeres are special chromatin structures at the ends of eukaryotic chromosomes, which protect from cellular senescence and apoptosis induced by genomic instability [2, 3]. The shortening of leukocyte telomere length (LTL) and the risk of T2DM has attracted increasing interest recently, but with inconsistent results [4–9]. The reasons might relate by the variability in population and measurement of LTL, as well as the cumulative effect of environmental and behavioral exposures [10, 11].

DNA methylation has been involved in the pathogenesis of a variety of biological processes and is affected by environmental factors as well as aging [12, 13]. Increasing evidence indicates that DNA methylation is involved in the regulation of mammalian telomeres, which could in turn control telomere length and function [14–16]. LINE-1, the only active long interspersed element (LINE), is a highly repeated sequence that constitutes about approximately 17% of the human genome [17]. Quantifying LINE-1 methylation is thought to serve as a surrogate measurement of global DNA methylation level [18, 19].

Previous studies has shown that epigenetics, especial for DNA methylation underlies gene expression and plays an essential role in the growing incidence of T2DM [12, 13, 20, 21].Thus, we conducted this case–control study to investigate the cross-talking of LTL shortening and LINE-1 DNA methylation in the risk of T2DM, and found the shortening of LTL related to DNA hypermethylation of LINE-1, which might provide new insights into the pathways underlying the association between telomere and T2DM.

## RESULTS

### General characteristics of subjects

Table 1 depicted the baseline characteristics of all participants. Significant shorter LTLs were found in T2DM comparing with controls (0.94 ± 0.41 vs. 1.14 ± 0.48, *P* < 0.001) while the ratio of LINE-1 DNA methylation was significantly elevated in T2DM compared with controls (49.60 ± 14.55% vs. 37.81 ± 9.07%, *P* < 0.001). Moreover, the T2DM group had significantly high levels of fasting blood glucose (FBG), glycated hemoglobin (HbA1c), and high-sensitivity C-reactive protein (hs-CRP) but low of total cholesterol (TC), high density lipoprotein cholesterol (HDL-C), low density lipoprotein cholesterol (LDL-C), apolipoprotein A1 (ApoA1) and apolipoprotein B (ApoB).

**Table 1:** Clinical characteristics of patients with type 2 diabetes mellitus (T2DM) and control subjects

Clinical characteristics	Control subjects (*n =* 213)	T2DM patients (*n =* 205)	*P* value
Age (years)	59.46 ± 8.74	61.22 ± 10.54	0.063
Male (%)	100 (46.95)	104 (50.73)	0.439
FBG (mmol/l)	5.34 ± 0.45	8.21 ± 2.24	**< 0.001**
HbA1c (%)	5.57 ± 0.36	7.52 ± 1.33	**< 0.001**
LTL (T/S ratio)	1.14 ± 0.48	0.94 ± 0.41	**< 0.001**
LINE-1 methylation (%)	37.81 ± 9.07	49.60 ± 14.55	**< 0.001**
TC (mmol/L)	5.01 ± 0.96	4.62 ± 1.14	**< 0.001**
TG (mmol/L)	1.46 ± 0.85	1.63 ± 1.05	0.072
HDL-C (mmol/L)	1.37 ± 0.37	1.18 ± 0.31	**< 0.001**
LDL-C (mmol/L)	3.23 ± 0.88	2.76 ± 0.94	**< 0.001**
ApoA1(g/l)	1.43 ± 0.27	1.32 ± 0.23	**0.007**
ApoB (g/l)	1.04 ± 0.26	0.95 ± 0.27	**0.038**
hs-CRP (ng/mL)	1.33 ± 2.57	2.04 ± 1.88	**0.026**
Hcy (μmol/l)	17.36 ± 14.80	15.91 ± 8.67	0.621
SBP (mmhg)	128 ± 19.45	134 ± 20.42	0.058
DBP (mmhg)	77 ± 11.09	76 ± 11.08	0.654
BMI	24.94 ± 3.49	25.84 ± 3.88	0.093

Data are presented as *n* (%) or mean ± standard deviation (SD). LTL (T/S ratio), ratio of telomere repeat copy number (T) to single-copy gene copy number (S); FBG, fasting blood glucose; HbA1c, glycated hemoglobin; TC, Total cholesterol; TG, Triglyceride; HDL-C, High density lipoprotein cholesterol; LDL-C, Low density lipoprotein cholesterol; ApoA1, apolipoprotein A1; ApoB, apolipoprotein B; hs-CRP, high-sensitivity C-reactive protein; Hcy, homocysteine; SBP, Systolic blood pressure; DBP, Diastolic blood pressure; BMI, Body mass index. The Bold = Significant Difference.

### Correlation between LTL and clinical characteristics

**Figure 1:**
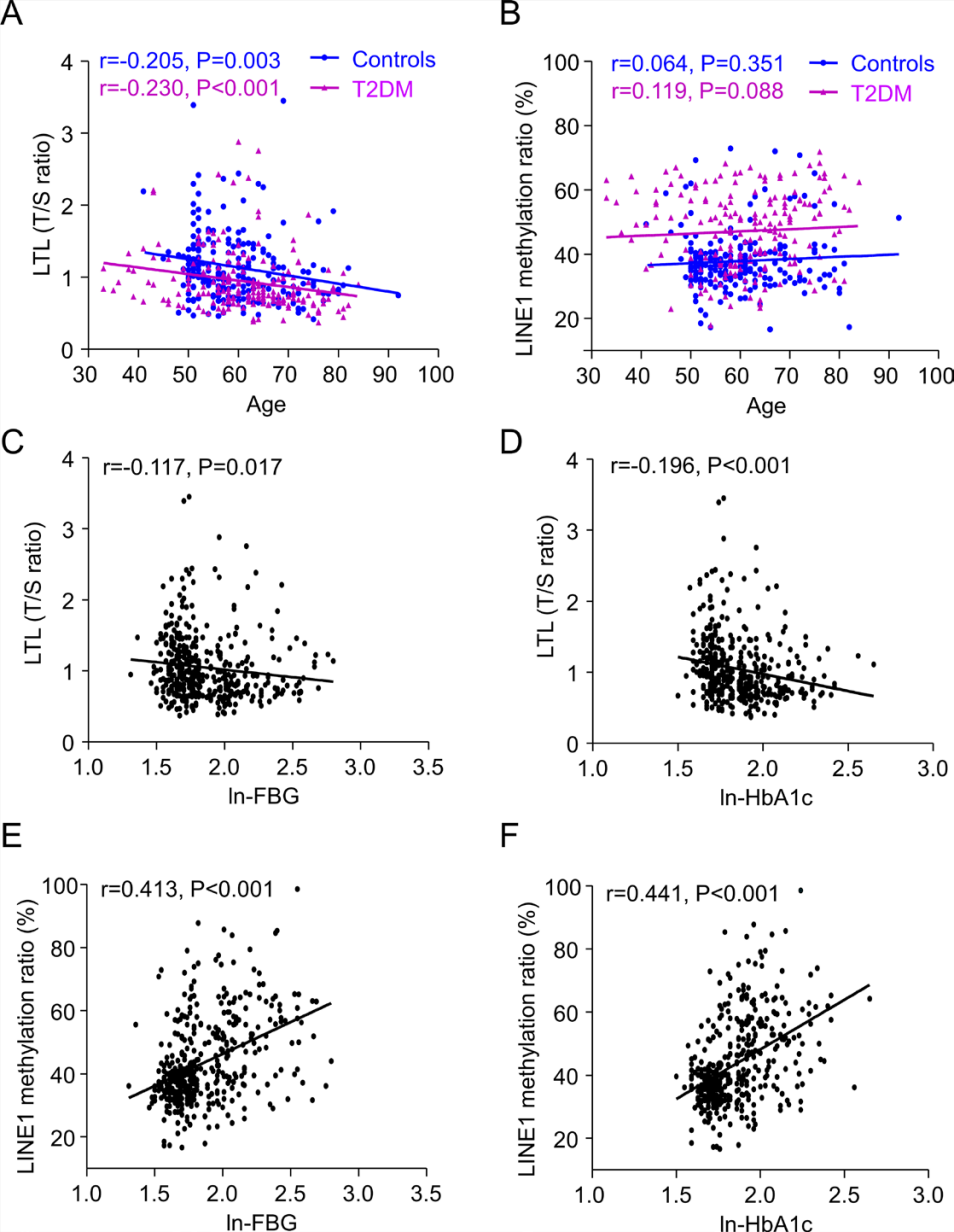
Linear regression analysis of the association between leukocyte telomere length (LTL) (A) and LINE-1 methylation ratio (B) with age in controls and T2DM patients; Controls are shown as blue dots (*n* = 213), and T2DM patients as purple triangles (*n* = 205). As well as correlation of LTL with ln-FBG (C) and ln-HbA1c (D), and LINE-1 methylation ratio with ln-FBG (E) and ln-HbA1c (F). LTL is plotted as T/S ratio (ratio of the copy number of telomere (T) repeats to that of a single (S) gene). FBG and HbA1c values were ln-transformed and described as ln-FBG and ln-HbA1c to normalize the distributions with no skew.

LTL (T/S ratio) did not differ between females and males (1.03 ± 0.42 vs. 1.06 ± 0.49, *P* = 0.652) but decreased steadily with age in both T2DM and controls, respectively (*r* = –0.230, *P* < 0.001; *r* = –0.205, *P* = 0.003; Figure 1A). LTL decreased with both ln-FBG and ln-HbA1c in all subjects after adjustment for age and sex (*r* = –0.117, *P* = 0.017; *r* = –0.196, *P* < 0.001; Figure 1C, 1D). Conversely, the ratio of LINE-1 DNA methylation were not related to age in both T2DM and controls (*r* = 0.119, *P* = 0.088; *r* = 0.064, *P* = 0.351; Figure 1B). No difference was found between females and males in LINE-1 methylation (44.08 ± 13.20 vs. 43.08 ± 13.68, *P* = 0.446). Inversely, ln-FBG (*r* = 0.413, *P* < 0.001; Figure 1E) and ln-HbA1c (*r* = 0.441, *P* < 0.001; Figure 1F) were positively correlated with LINE-1 DNA methylation ratio in all participants adjusted for age and sex.

### LTL associated with LINE-1 methylation and HbA1c

We further analyzed the relationship between LTL and LINE-1 DNA methylation ratio, and biochemical parameters adjusted for age and sex. As shown in Table 2, univariate linear regression showed that LINE-1 DNA methylation ratio, HbA1c, FBG, and LDL-C were found to significantly correlate with LTL. After multi-adjustment, age [regression coeffiecnt (95% CI) = −0.010 (−0.014 to −0.005), *P* < 0.001], HbA1c [−0.046 (−0.091 to 0.000), *P* = 0.048] and LINE-1 methylation ratio [−0.008 (−0.012 to −0.005), *P* < 0.001] were stably negatively associated with LTL (Table 3).

**Table 2:** Univariate linear regression analyses of the relationship between clinical characteristics and LTL (T/S ratio) adjusted for age and sex in all participants

Clinical characteristics	LTL (T/S ratio) age and sex-adjusted		
	B	95% CI	*P*
LINE-1 methylation (%)	−0.009	−0.012 to −0.006	**< 0.001**
HbA1c (%)	−0.060	−0.091 to –0.029	**< 0.001**
FBG (mmol/l)	−0.022	−0.042 to −0.002	**0.034**
TC (mmol/L)	0.030	−0.012 to 0.071	0.160
TG (mmol/L)	0.019	−0.027 to 0.064	0.424
HDL-C (mmol/L)	0.018	−0.109 to 0.145	0.781
LDL-C (mmol/L)	0.054	0.008 to 0.101	**0.022**
ApoA1 (g/l)	0.111	−0.118 to 0.340	0.340
ApoB (g/l)	−0.039	−0.239 to 0.161	0.701
hs-CRP (ng/mL)	−0.021	−0.048 to 0.005	0.152
Hcy (μmol/l)	−0.003	−0.010 to 0.003	0.221
SBP (mmhg)	−0.001	−0.004 to 0.003	0.856
DBP (mmhg)	−0.001	−0.006 to 0.005	0.865
BMI	0.002	−0.015 to 0.018	0.571

LTL (T/S ratio), ratio of telomere repeat copy number (T) to single-copy gene copy number (S); HbA1c, glycated hemoglobin; FBG, fasting blood glucose; TC, Total cholesterol; TG, Triglyceride; HDL-C, High density lipoprotein cholesterol; LDL-C, Low density lipoprotein cholesterol; ApoA1, apolipoprotein A1; ApoB, apolipoprotein B; hs-CRP, high-sensitivity C-reactive protein; Hcy, homocysteine; SBP, Systolic blood pressure; DBP, Diastolic blood pressure; BMI, Body mass index. The Bold = Significant Difference.

**Table 3:** Multivariate linear regression analysis of the relationship between clinical characteristics and LTL (T/S ratio) in all participants

Clinical characteristics	LTL (T/S ratio) multi-adjusted		
	B	95% CI	*P*
Age	−0.010	−0.014 to −0.005	**< 0.001**
Sex	0.029	−0.056 to 0.113	0.504
LINE-1 methylation (%)	−0.008	−0.012 to −0.005	**< 0.001**
HbA1c (%)	−0.046	−0.091 to 0.000	**0.048**
FBG (mmol/l)	0.021	−0.008 to 0.049	0.158
LDL-C (mmol/L)	0.021	−0.026 to 0.068	0.382

LTL (T/S ratio), ratio of telomere repeat copy number (T) to single-copy gene copy number (S); HbA1c, glycated hemoglobin; FBG, fasting blood glucose; LDL-C, Low density lipoprotein cholesterol. The Bold = Significant Difference.

### Association between LINE-1 methylation and clinical characteristics

Table 4 presented the relationship between LINE-1 methylation and clinical characteristics adjusted for age and sex in all participants. The results of univariate linear regression revealed that increased LINE-1 methylation ratio was associated with increasing HbA1c [0.043 (0.034 to 0.052), *P* < 0.001] and FBG [0.063 (0.048 to 0.077), *P* < 0.001]. That was to say, every 10% increase in LINE-1 DNA methylation, HbA1c, FBG increased by 0.43, 0.63 mmol/l, respectively. As for lipid biomarkers, LINE-1 DNA methylation was negatively associated with TC [−0.016 (−0.024 to −0.009), *P* < 0.001], HDL-C [−0.005 (−0.008 to −0.003), *P* < 0.001], LDL-C [−0.019 (−0.025 to −0.012), *P* < 0.001], ApoB [−0.004 (−0.006 to −0.001), *P* = 0.006] which meant TC, HDL-C, LDL-C, ApoB decreased by 0.16, 0.05, 0.19 and 0.04 mmol/l following 10% increase in LINE-1 DNA methylation, respectively. No association was found between methylation of LINE-1 and hcy, blood pressure and BMI. Considering the high degree correlation between the variables, multivariable linear model was not conducted.

**Table 4:** Univariate linear regression analyses of relationships between LINE-1 methylation ratio and the listed dependent variables adjusted for age and sex in all participants

Clinical characteristics	LINE-1 methylation ratio by age and sex-adjusted		
	B	95% CI	*P*
HbA1c (%)	0.043	0.034 to 0.052	**< 0.001**
FBG (mmol/l)	0.063	0.048 to 0.077	**< 0.001**
TC (mmol/L)	−0.016	−0.024 to −0.009	**< 0.001**
TG (mmol/L)	0.001	−0.005 to 0.008	0.685
HDL-C (mmol/L)	−0.005	−0.008 to −0.003	**< 0.001**
LDL-C (mmol/L)	−0.019	−0.025 to −0.012	**< 0.001**
ApoA1 (g/l)	−0.002	−0.005 to 0.000	0.053
ApoB (g/l)	−0.004	−0.006 to −0.001	**0.006**
Hcy (μmol/l)	−0.251	−0.579 to 0.077	0.132
SBP (mmhg)	−0.245	−0.512 to 0.021	0.071
DBP (mmhg)	−0.018	−0.173 to 0.138	0.824
BMI	0.008	−0.039 to 0.056	0.732

HbA1c, glycated hemoglobin; FBG, fasting blood glucose; TC, Total cholesterol; TG, Triglyceride; HDL-C, High density lipoprotein cholesterol; LDL-C, Low density lipoprotein cholesterol; ApoA1, apolipoprotein A1; ApoB, apolipoprotein B; Hcy, homocysteine; SBP, Systolic blood pressure; DBP, Diastolic blood pressure; BMI, Body mass index. The Bold = Significant Difference.

### Short LTL, LINE-1 hypermethylation increase the risk of T2DM

In age-sex adjusted logistic regression analyses shorter LTL was associated with an increased risk of T2DM (lowest *vs.* highest quartile: OR = 2.828, 95% CI 1.460–5.477, *P* = 0.002; P for trend over quartiles < 0.001). The results remained similar in multivariable-adjusted models accounting for TC, TG, HDL-C, LDL-C. However, the pattern of association was inverse in LINE-1 methylation. Lower LINE-1 methylation ratio was found significantly associated with a reduced risk of T2DM (lowest *vs.* highest quartile: OR = 0.1, 95% CI 0.05–0.20, *P* < 0.001; P for trend over quartiles < 0.001). Additional adjustment for biomarkers showed a similar pattern of association. The detailed results were shown in Table 5.

**Table 5:** Logistic regression analyses of the association between LTL, LINE-1 methylation ratio and T2DM in all participants

Clinical Characteristics	Age and sex-adjusted model			Multivariable-adjusted model^a^		
	OR (95% CI)	*P*	*P*_trend_	OR (95% CI)	*P*	*P*_trend_
LTL (T/S ratio)						
Q1	2.828 (1.460, 5.477)	**0.002**		2.458 (1.192, 5.070)	**0.015**	
Q2	1.946 (1.023, 3.700)	**0.042**		1.738 (0.868, 3.478)	0.119	
Q3	1.145 (0.613, 2.137)	0.671		1.247 (0.640, 2.430)	0.516	
Q4	Reference	-	**< 0.001**	Reference	-	**< 0.001**
LINE-1 methylation (%)						
Q1	0.1 (0.050, 0.200)	**< 0.001**		0.189 (0.089, 0.400)	**< 0.001**	
Q2	0.084 (0.042, 0.168)	**< 0.001**		0.158 (0.073, 0.340)	**< 0.001**	
Q3	0.258 (0.133, 0.499)	**< 0.001**		0.379 (0.185, 0.779)	**0.008**	
Q4	Reference	-	**< 0.001**	Reference	-	**< 0.001**

LTL (T/S ratio), ratio of telomere repeat copy number (T) to single-copy gene copy number (S); OR, odds ratio; CI, confidence interval, both estimated from logistic regression analysis.

^a^Adjusted for age, sex, total cholesterol, triglyceride, high density lipoprotein cholesterol, low density lipoprotein cholesterol.

The Bold = Signifcant Difference.

## DISCUSSION

Our current case–control study findings show the following: 1) T2DM has shortened LTL and hypermethylation of LINE-1; 2) LINE-1 hypermethylation, aging and HbA1c may contribute to the process of telomere shortening in T2DM; 3) LINE-1 hypermethylation may increase the level of HbA1c and FBG; 4) Short LTL and LINE-1 hypermethylation increase the risk of T2DM.

LTL is affected by the accumulated burden of environmental exposure and genetic predisposition [2,3]. During the past decade, an increasing number of studies have been carried out to clarify the relationship between telomere length and T2DM, but the evidence is not consistent [4–7, 9, 22–27]. Most studies have indicated the association between the shortening of LTL and T2DM [6, 7, 9, 23–27]. Indeed, two recent meta-analyses similarly concluded that telomere shortening was a significant risk factor for T2DM [28, 29]. However, in a population-based study of 3921 participants in the U.S., no association was found between LTL and diabetes status, diabetes duration, or diabetes control [5]. Considering the ethnic differences, the result may be not suitable for the general population. Our current study included 418 cases of Chinese aged 30–90 years and we found that LTL was significantly shortened in T2DM compared with controls, which was consistent with previous studies in Asian populations [6, 7, 24, 25, 30] implying the interaction between shortened telomere length and T2DM. No correlation was found between gender and LTL. Nevertheless, like other cross-sectional studies, our study could not explain whether the shortened telomere length in T2DM was a cause or a consequence.

Previous studies also suggested an association between DNA methylation and telomere length [31, 32]. However, no attention was focused on the relationship between DNA methylation and telomere length in the pathophysiology of T2DM. Herein, we further explored the pattern of LINE-1 methylation in T2DM and illustrated the association between LTL and methylation.

Our study showed LINE-1 was significantly hypermethylated in T2DM patients, lower LINE-1 DNA methylation levels were associated with a reduced risk of T2DM. Different from other studies, no correlation was found between age, gender and LINE-1 methylation [33, 34]. Additionally, DNA methylation changes are indicated to involve in the development of some complex diseases and might be useful in predicting the risk of T2DM and CVD. However, some controversies still remain. Several studies reported global DNA methylation was associated with altered levels of lipid profiles, however, the patterns of dynamic changes of lipid levels were inconsistent [35–37]. *Pearce* et al. has shown an increase of global DNA methylation measured in LINE-1 with increasing FBG in 228 participants from northern England [35]. Similarly, blood glycaemic variables HbA1c, FBG were both positively associated with DNA methylation in our study. After correcting the lipid biomarkers, LINE-1 hypermethylation was still a risk factor of T2DM. The result was also supported by *Ling*, his team found the PPARGC1A gene promoter was hypermethylated in diabetic islets compared with non-diabetic islets [38], while some studies also indicated the association between T2DM and DNA hypomethylation [34, 36]. The inconsistent results of LINE-1 DNA methylation patterns may due to some confounding such as race, underlying diseases, or the association is not causally associated to diabetes.

What’s more, the level of LINE-1 methylation was stably negatively related with peripheral blood LTL. Unlike tandem DNA repeats, DNA methylation of LINE-1 was very consistent among individual persons [39], which has been widely used as a surrogate measurement of global DNA methylation level [18, 19]. The telomeric and subtelomeric regions are both enriched in repetitive DNA with abundant epigenetic prints. In the subtelomeric region, LINEs are over-represented by accounting for approximately 25% of the number of bases, which is higher than that of genome [40]. High percentage of LINE-1 may somewhat account for the association between methylation levels of subtelomeric regions and telomere length. Therefore, it is reasonable to postulate that LINE-1 hypermethylation, accompanying with aging and HbA1c may collectively contribute to the process of telomere shortening in T2DM.

Recently, several studies have investigated the association between DNA methylation of LINE-1 and telomere length [41–43], but with inconsistent results. In our study, LINE-1 DNA hypermethylation was associated with the shortening of LTL in T2DM. This is supported by a previous *in vitro* study that, demonstrated a functional relationship between DNA hypomethylation and increased telomeric recombination in cells lacking DNA methyltransferases (DNMTs) [14]. Further evidence from a cohort study indicated a negative association between LINE-1 methylation and LTL in an elderly cohort with a supplement of vitamin B [44]. LINE-1 hypomethylation has also been linked to telomere shortening in Wilms tumor [42]. The inconclusiveness of the association between telomere length and LINE-1 DNA methylation might relate to different diseases, populations and methodology; further investigations are required.

However, limitations inevitably existed in our study. The current findings were limited to Chinese subjects; whether they fit for other populations needs further discussion. As some potential confounders such as smoking, physical activity were not measured, bias could exist regarding the observed results. Moreover, the duration of diabetes and the effect of antidiabetic drugs on telomere length were not considered. As a great amount of T2DM has a silent onset, specific age of onset is difficult to define. Likewise, calculating the effect of drugs could be difficult due to the uneven time of treatment and inconsistent use of medicine. However, no explicit relationship between antidiabetic drugs and telomere length is reported, and no association was found between LTL and diabetes duration [5]. Our retrospective study explicated the cross-sectional relationship between telomere length and DNA methylation in T2DM, which might provide valuable information regarding possible confounders in telomere dynamics among humans.

## MATERIALS AND METHODS

### Participants

This was a random hospital-based case-control study including 205 T2DM patients consecutively attending the outpatient of Peking Union Medical College Hospital, China. A total of 213 subjects were selected, matched for sex and age with healthy subjects who were visiting the hospital for a health examination. All patients were between 30 and 90 years of age and met the following criteria: 1) have a previous diagnosis of diabetes, glycated hemoglobin (HbA1c) ≥ 6.5% or fasting blood glucose (FBG) ≥ 126 mg/dl (7 mmol/l), which was in accordance with the 2015 American Diabetes Association criteria [45]; 2) free of severe systemic diseases and communicable diseases, including immune system diseases, cancer, tuberculosis and AIDS ; 3) no diagnosis of type 1 diabetes mellitus or secondary forms of diabetes. Height, weight, and blood pressure were measured in all participants. All healthy controls had no history of diabetes mellitus, HbA1c < 6.5% and fasting blood glucose (FBG) < 110 mg/dl (6.1 mmol/l). All participants were required to give informed consent in accordance with the Helsinki Declaration. All of the experimental protocols were approved by the Ethics Committee of Peking Union Medical College Hospital. The methods were carried out in accordance with the relevant guidelines.

### Clinical laboratory tests

Fasting venous blood was drawn from all subjects into EDTA vacutainers. After centrifugation, the serum and leukocyte cells were separated into new tubes for biochemical measurements and DNA analysis, respectively, and stored at –80°C until used. Biochemical variables, including serum total cholesterol (TC), triglycerides (TG), high-density lipoprotein cholesterol (HDL-C), and low-density lipoprotein cholesterol (LDL-C) were measured with a Beckman AU Series Automatic Biochemical Analyzer (Japan), using Sekisui Medical (Japan) reagents. FBG, apolipoprotein A1 (ApoA1), apolipoprotein B (ApoB), and high-sensitivity C-reactive protein (hs-CRP) were measured with the same instrument, using Beckman AU reagents, and homocysteine (Hcy) was examined using Beijing Leadman reagents. HbA1c was examined by high-performance liquid chromatography (HPLC) on a Bio-Rad Diamat automated glycosylated hemoglobin analyzer (USA). Coefficients of variation were < 10% for Hcy, hs-CRP, and < 5% for all other variables.

### LTL determination

DNA samples were extracted using the TIANamp Genomic DNA kit (Beijing), and quantified using Nanodrop-2000 (NanoDrop Technologies). Leukocyte telomere length was measured by a quantitative PCR (qPCR) method as previously described [46] which compares the ratio of the telomere repeat copy number (T) to the single-copy gene copy number (S) expressed as the telomere length ratio (T/S ratio). Telomere and 36B4 primer sequences were: telomere upstream: 5′-GGTTTTTGAGGGTGAGGGTGAGGGTGAGGGTGAGGGT-3′; telomere downstream: 5′-TCCCGACTATCCCTATCCCTATCCCTATCCCTATCCCTA-3′; 36B4 upstream, 5′-CAGCAAGTGGGAAGGTGTAATCC-3′; 36B4 downstream, 5′-CCCATTCTATCATCAACGGGTACAA-3′. All samples were amplified with the use of the LightCycler 480 (Roche, Switzerland) and measured in triplicate. 293T cells were used for reference DNA samples and measured for telomere length ratios by dilution series from 1.56 to 100.00 ng (2-fold dilution; 7 points) [32, 47]. Correlation of telomeres and β-globin qPCR was linear (R^2^ = 0.98).

### Quantitative of LINE-1 DNA methylation

As we previously described [47], blood genome DNA was modified by BisulFlash DNA Modification kit (Epigentek Group Inc., NY, USA). Quantitative methylation-specific PCR (qMSP) was performed with a primer set specific to methylated or unmethylated sequence by bisulfite-treated DNA and conducted by the LightCycler 480 (Roche, Switzerland) for the measurement of LINE-1 DNA methylation. The LINE-1 primers used were: unmethylated forward primer, 5′-TGTGTGTGAGTTGAAGTAGGGT-3′, reverse primer, 5′-ACCCAATTT TCCAAATACAACCATCA-3′; methylated forward primer, 5′-CGCGAGTCGAA GTAGGGC-3′, reverse primer, 5′-ACCCGATTTTCCAAATACGACCG-3′. Human methylated and non-methylated DNA sets (Epigentek Group Inc., NY, USA) were used as negative and positive controls for detecting methylation ratio and drawing standard curve. Each sample was measured in triplicate. The methylation levels were calculated as the fraction of methylated molecules in the total number of DNA molecules (the number of methylated molecules + the number of unmethylated molecules) [48].

### Statistical analysis

Continuous variables with a normal distribution were described as the mean ± standard deviation (SD) and analyzed by unpaired *t*-test. Sex was analyzed as percentages, using the *chi*-squared test. The correlation between LTL, LINE-1 methylation ratio, ln-FBG, and ln-HbA1c were analyzed by Pearson correlation coefficient with adjustment for age and sex. Univariate linear regression analysis was applied to examine potential association between each factor and LTL, LINE-1 methylation ratio. In multivariate analyses, all statistically significant factors were adjusted as potential confounding variables, and the regression coefficients and 95% CIs were calculated. The associations between the LTL, LINE-1 methylation and T2DM were measured by calculating odd ratios (ORs) and 95% confidence intervals (CIs) using logistic regression. Statistical significance was assumed at *P* < 0.05, and all analyses were conducted using SPSS 16.0 (SPSS Inc., Chicago, IL, USA).

## CONCLUSIONS

Our findings indicate that aging, LINE-1 methylation, and HbA1c might collectively contribute to shortening of LTL in subjects with T2DM. Shorter LTL was associated with an increased risk of T2DM, while lower LINE-1 DNA methylation levels could reduce the risk of T2DM. These findings provide novel insights into an epigenetic mechanism for shortened LTL in DM. However, larger, well-designed studies are needed to confirm these findings and explore sources of heterogeneity.

## Abbreviations

ApoA1: Apolipoprotein A1
ApoB: Apolipoprotein B
BMI: Body mass index
DBP: Diastolic blood pressure
FBG: Fasting blood glucose
HbA1c: Glycated hemoglobin
Hcy: Homocysteine
HDL-C: High density lipoprotein cholesterol
hs-CRP: High-sensitivity C-reactive protein
LDL-C: Low-density lipoprotein cholesterol
LINE: Long interspersed element
LTL: Leukocyte telomere length
SBP: Systolic blood pressure
T2DM: Type 2 diabetes
TC: Total cholesterol
TG: Triglycerides
